# Ductal Carcinoma In Situ (DCIS) and Microinvasive DCIS: Role of Surgery in Early Diagnosis of Breast Cancer

**DOI:** 10.3390/healthcare11091324

**Published:** 2023-05-05

**Authors:** Francesca Magnoni, Beatrice Bianchi, Giovanni Corso, Erica Anna Alloggio, Susanna Di Silvestre, Giuliarianna Abruzzese, Virgilio Sacchini, Viviana Galimberti, Paolo Veronesi

**Affiliations:** 1 Division of Breast Surgery, European Institute of Oncology (IEO), IRCCS, 20141 Milan, Italy; beatrice.bianchi@ieo.it (B.B.); giovanni.corso@ieo.it (G.C.); ericaanna.alloggio@ieo.it (E.A.A.); giuliarianna.abruzzese@ieo.it (G.A.); viviana.galimberti@ieo.it (V.G.); paolo.veronesi@ieo.it (P.V.); 2 European Cancer Prevention Organization (ECP), 20141 Milan, Italy; 3 Department of Oncology and Hemato-Oncology, University of Milan, 20122 Milan, Italy; 4 Breast Service, Department of Surgery, Memorial Sloan Kettering Cancer Center, New York, NY 10065, USA; sacchinv@mskcc.org

**Keywords:** breast cancer, prevention, ductal carcinoma in situ, DCIS, microinvasion, microinvasive breast cancer, breast surgery, quality of life

## Abstract

Advances in treatments, screening, and awareness have led to continually decreasing breast cancer-related mortality rates in the past decades. This achievement is coupled with early breast cancer diagnosis. Ductal carcinoma in situ (DCIS) and microinvasive breast cancer have increasingly been diagnosed in the context of mammographic screening. Clinical management of DCIS is heterogenous, and the clinical significance of microinvasion in DCIS remains elusive, although microinvasive DCIS (DCIS-Mi) is distinct from “pure” DCIS. Upfront surgery has a fundamental role in the overall treatment of these breast diseases. The growing number of screen-detected DCIS diagnoses with clinicopathological features of low risk for local recurrence (LR) allows more conservative surgical options, followed by personalised adjuvant radiotherapy plans. Furthermore, studies are underway to evaluate the validity of surgery omission in selected low-risk categories. Nevertheless, the management, the priority of axillary surgical staging, and the prognosis of DCIS-Mi remain the subject of debate, demonstrating how the paucity of data still necessitates adequate studies to provide conclusive guidelines. The current scientific scenario for DCIS and DCIS-Mi surgical approach consists of highly controversial and diversified sources, which this narrative review will delineate and clarify.

## 1. Introduction

The World Health Organization (WHO) has defined two distinct but connected strategies to favour precocious cancer detection and early diagnosis—the recognition of symptomatic cancer at an early stage, and screening, which is the identification of asymptomatic disease in a target population of apparently healthy individuals [1,2]. Within this framework, efforts to promote early detection are prerequisites for population-based screening that will improve outcomes for all breast cancer (BC) patients.

Early detection programs, part of a comprehensive health care system, and various international research consortia working on different aspects of the personalised BC early detection and prevention (for instance, European Collaborative on Personalized Early Detection and Prevention of Breast Cancer-ENVISION-) [3] allow for the facilitation of treatments of initially diagnosed breast cancers, by containing their burden and mitigating their adverse effects. Almost half of the reduction in BC mortality is due to efforts in prevention and early diagnosis [4,5]. Nonetheless, advances in systemic treatments, targeted monoclonal antibodies, immunotherapy, and gene expression profiling, have become predominant since the early 2000s and are still effective [6]. 

Precocious diagnosis increasingly requires clinicians to better manage early breast diseases, such as ductal carcinoma in situ (DCIS), or DCIS with microinvasion (DCIS-Mi). 

**Figure 1:**
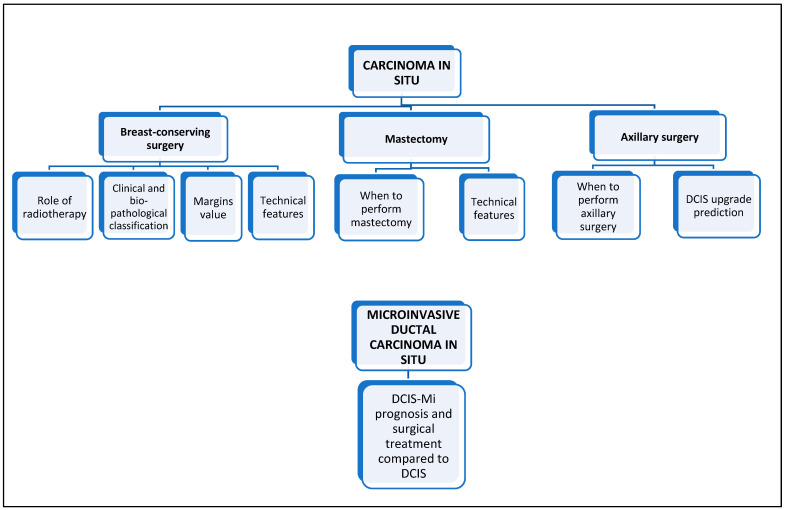
Review workflow. DCIS—ductal carcinoma in situ; DCIS-Mi—microinvasive DCIS.

In this era of precision medicine, increasingly sensitive to the impact of treatments on women’s quality of life, the role of surgery in the overall treatment of DCIS and DCIS-Mi is a dynamic and diverse issue. This review, therefore, focuses on analysing the de-escalation in the surgical approach to these clinical conditions in its different modalities, exploring the current and future potential de-implementations (See Figure 1).

## 2. Method

The searching strategy involved PUBMED database analysis, using the terms ‘ductal carcinoma in situ’, ‘DCIS’, ‘ductal carcinoma in situ with microinvasion’, ‘microinvasive breast cancer’, ‘margins’, ‘prognosis’, ‘surgery’, ‘mastectomy’, ‘breast conserving surgery’, and ‘sentinel node biopsy’ from 1999 to present, focusing on randomized clinical trials, original articles, reviews, meta-analyses and observational/retrospective studies of last two decades, including these selected keywords. Full-text available articles published in the English language were included. Each selected article was critically evaluated according to the key findings, limitations, propriety of the methods used to verify the initial hypothesis, quality and interpretation of the results obtained, as well as to the impact of the conclusions.

## 3. Ductal Carcinoma In Situ

Ductal carcinoma in situ is a group of diversified premalignant breast lesions, representing a nonmandatory precursor of invasive BC [7]. With the widespread application of population-based BC screening programs and digitalised imaging, the incidence of DCIS has progressively increased and now accounts for 20 to 25% of newly diagnosed breast cancers [8,9,10]. Mammography for BC screening detects around 35% of DCIS in asymptomatic women [11]. According to the American Cancer Society, over 50,000 cases of female breast ductal carcinoma in situ diagnosed in 2022 have been predicted in the United States [12]. Because of the complexity and polyhedrality of DCIS lesions in terms of their biological and pathological features, uncertainty exists regarding how and which DCIS lesions will develop into invasive cancer, and several models have been proposed to explain this progression [13]. However, at present, all out-of-trial patients diagnosed with breast DCIS are initially treated with surgery, followed by adjuvant treatments, including radiotherapy or hormone therapy, if needed. Considering that patients diagnosed with ductal carcinoma in situ have a reported excellent 10-year overall survival (80–90%) [14], a high rate of overtreated patients could benefit from less aggressive treatments. Albeit a specific subgroup of patients who could be appropriately treated with excision alone is yet to be identified, the risk of overtreatment is the primary concern since a proportion of DCIS never progresses to invasive BC. Nowadays, there is a growing impulse to unburden the management of ductal carcinoma in situ, including a possible nonoperative approach for low-grade and intermediate-grade DCIS (low-risk DCIS). Indeed, several clinical trials are looking at the decision-making process of low-risk DCIS patients with active surveillance instead of traditional treatment, such as Comparison of Operative versus Monitoring and Endocrine Therapy (COMET), the United States, which is planned to complete accrual by July 2023 [15,16], (LORIS) United Kingdom [17], (LORD) Europe [18], and (LORETTA) Japan [19]. 

On the other side, studies have found that active surveillance would lead to undertreatment of up to 24% of patients using the current eligibility criteria [9]; approximately one in five patients with low-risk DCIS presented invasive disease at the time of surgery, making active surveillance a not-risk-free option [20]. Stricter criteria would be necessary to improve the selection of patients with low-risk DCIS. Available evidence supports the role of nomograms and gene expression profile studies in guiding ductal carcinoma in situ clinical management [21], emphasising that a de-escalation approach also involves DCIS patient care. Again, in the context of early breast disease, the purpose of multidisciplinary clinical management is to achieve an individualised therapy to attenuate treatment-related morbidities, preserving the quality of life while balancing oncologic and cosmetic outcomes. 

This purpose is captured in a new landscape, unifying each theoretical criterion into homogeneous clinical practical applications shared by all the cancer centres worldwide. 

### 3.1. Breast-Conserving Surgery

**Figure 2:**
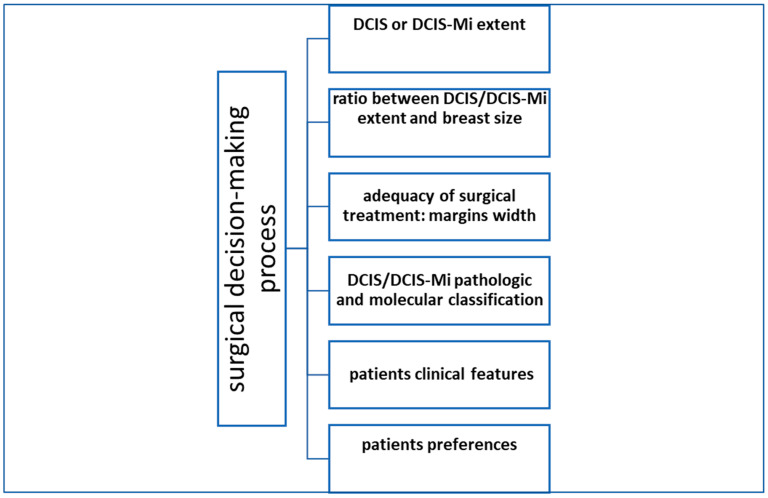
Clinical features impacting on DCIS/DCIS-Mi surgical decision-making process. DCIS—ductal carcinoma in situ; DCIS-Mi—microinvasive DCIS.

In this kaleidoscopic landscape in which the concept of “in-breast recurrence” risk [22] in ductal carcinoma in situ is widely studied, the role of breast-conserving surgery (BCS) is closely connected to those of radiotherapy and endocrine therapies. As randomized trials show [23], a class of DCIS with an absolute moderate risk for LR could be identified. Bearing in mind that the whole treatment for DCIS does not impact a survival benefit, the 2019 Saint Gallen Consensus [24] defined specific favourable prognostic parameters (i.e., low- or intermediate-grade absence of comedonecrosis, age > 50 years, as well as broad surgical margins, generally greater than 0.5 cm) on the basis of which women might waive radiotherapy and endocrine treatments. Thus, the proper diagnostic definition of ductal carcinoma in situ extent detection is essential (see Figure 2). The additional diagnostic role of magnetic resonance (MRI) is controversial [25]. MRI was reported to have a sensitivity of up to 98% for high-grade DCIS [26], but in contrast, some studies have suggested that it can overestimate the extent of the disease [27]. The NCCN Panel recommends only performing breast MRI for DCIS in selected cases, given that the use of MRI has not been shown to increase the probability of negative margins or decrease conversion to mastectomy for DCIS [12]. Ductal carcinoma in situ itself is not associated with lymph/angioinvasion and metastasis. Similarities in morphology and genetic profiles between invasive and in situ breast cancers indicate that DCIS is a precursor to invasive carcinoma [28]. Moreover, it is currently a matter of controversy and debate which DCIS lesion will progress to invasive cancer and which will persist with an inactive biological behaviour. DCIS survival is excellent, with a 20-year actuarial BC-specific mortality rate of 3.8% [9,28]. However, it has a 5 to 10 times higher risk of developing into invasive BC compared with the general population [22]. 

Hence, the prevention of DCIS development into an invasive carcinoma has become crucial in ductal carcinoma in situ management and treatment [22], with treatments aiming to prevent the subsequent development of invasive BC or DCIS recurrence, even though DCIS does not impact overall survival [28]. At present, most DCIS patients undergo surgery, historically one of the standard treatments for DCIS [21]. Concerning the size and extension of the lesion and in accordance with patient preferences, this approach can be conservative (quadrantectomy or lumpectomy) or radical (mastectomy). Until recently, mastectomy was the most effective surgical treatment in DCIS, as demonstrated in a comprehensive meta-analysis reporting a 1.4% local recurrence (LR) rate [29,30]. Several large-scale trials with long-term follow-up results have provided the cornerstone of the modern approach to the surgical treatment of early BC by demonstrating that BCS followed by radiotherapy offers oncological outcomes not inferior to mastectomy [31,32]. Currently, there are no studies comparing the outcome of BCS to that of mastectomy in the management of ductal carcinoma in situ.

#### 3.1.1. Role of Radiotherapy

The role of BCS associated with radiotherapy in managing DCIS has been extensively investigated, demonstrating its effectiveness of local control and confirming that BCS with radiotherapy represents the treatment of choice in the presence of limited ductal carcinoma in situ [33]. A meta-analysis of four large multicentre randomised trials demonstrated that the addition of radiotherapy after BCS for DCIS provides a statistically and clinically significant decrease in ipsilateral breast events (hazard ratio [HR], 0.49; 95% confidence interval [CI]; 0.41–0.58, *p* < 0.00001) [34]. However, these trials did not indicate that adding radiotherapy has an overall survival (OS) benefit. Indeed, several randomised trials compared the breast-conserving approach alone with the breast-conserving approach followed by radiation for DCIS. The National Surgical Adjuvant Breast and Bowel Project (NSABP) B-17 trial showed that radiation after BCS reduced invasive Ipsilateral Breast Tumour Recurrence (IBTR) by 52% compared with patients who did not receive adjuvant radiation. In addition, radiation provided a 10-year risk reduction in IBTR of 15% [35]. 

European Organization for Research and Treatment of Cancer (EORTC) 10,853 obtained similar findings of reduced rates of local recurrence resulting from adjuvant radiation after local excision of DCIS [36]. Randomised trials and meta-analyses [37,38] have shown that adjuvant radiotherapy reduces the rate of local recurrences (LRs) by about 50%, both for in situ and invasive forms. The highest risk of recurrence is associated with comedonecrosis, high-grade DCIS, positive or close to the margin, and young age. Subgroups of patients who do not benefit from adjuvant radiotherapy after conservative surgery have not yet been identified. 

The most recent randomised phase III study, NRG/RTOG 9804, which enrolled patients with low-risk DCIS (size ≤ 2.5 cm, margins ≥ 3 mm, and low or intermediate nuclear grade), also confirmed the persistence over time of the statistically significant reduction in LR (even invasive) in the radiotherapy arm compared with surgery alone, as well as a lengthening of the time of ductal carcinoma in situ reappearance [23]. Patients with the same eligibility characteristics as those in study NRG/RTOG 9804 may also be candidates to receive partial breast irradiation (APBI) following ASTRO Guidelines [39]. 

The omission of radiation treatment for in situ lesions is considered within the NCCN guidelines [12] as a personalised treatment in selected cases perceived to have a low risk of recurrence. The multidisciplinary discussion is crucial for a shared decision-making process, bearing in mind special factors such as age, lifetime expectancy, grading, margins status and whether the disease is endocrine-responsive. As the last St. Gallen Consensus Conference recommended, adding a radiotherapy boost decreases recurrence rates in non-low-risk DCIS cases [40].

#### 3.1.2. Clinical and Bio-Pathological Classification

Several scientific contributions have clarified the role of specific anatomical and biological factors involved in a possible DCIS LR (see Figure 2). The American Society for Clinical Oncology (ASCO)/CAP guidelines recommend oestrogen receptors (ER) testing of diagnosed ductal carcinoma in situ to determine the potential benefit of endocrine therapies for breast cancer risk reduction. [41]. Silverstein and colleagues have identified the Van Nuys Prognostic Index (VNPI) (histological type, width of surgical margin, and lesion size) as a selective tool to identify the class of patients at higher risk for invasive recurrence, even though ambiguity persists on which of these could be more significant [42]. In addition, recent research has validated the genetic contribution to the computation of recurrence risk: an ECOG 5194 DCIS observational study comprised a multigene expression assay to stratify patients into low- and high-risk groups [43]. 

The Oncotype DX DCIS score is a gene signature panel for DCIS patients that produces individualised estimates of the 10-year risk of any LR (DCIS or invasive) following BCS alone, especially in association with other clinical and pathological features, such as age, size, margin status, and multifocality, identifying those DCIS patients with a relatively low risk of recurrence who may support the omission of radiotherapy [43]. It is the only clinically validated and commercially available multigene prognostic signature considered a predictor of recurrence risk. It might be selectively used to guide a personalised recurrence risk assessment, although its clinical application does not represent a standard of care, and its routine use in clinical practice remains controversial, as it was not shown to be cost-effective [21]. Furthermore, a novel biosignature for identifying risk subtypes to help select patients with elevated breast recurrence risk after BCS plus radiotherapy has been evaluated in a recent study by Vicini and Colleagues [44]. Three different biosignatures were defined to assess the different degrees of risk for local recurrence and to select specific high-risk DCIS patients who can benefit from RT after BCS, likewise recognising a group of low-risk patients with excellent outcomes with BCS alone.

#### 3.1.3. Margins Value

The axiom “Less Is More, Bigger Is Not Better” [33] has influenced decades of invasive BC, as well as ductal carcinoma in situ surgical treatment, reinforcing the value of margin status in BCS since negative margins in DCIS are the most important local prognostic factor in breast-conserving therapy (BCT), i.e., BCS plus radiotherapy. The Society of Surgical Oncology (SSO), American Society for Radiation Oncology (ASTRO), and American Society of Clinical Oncology (ASCO) multidisciplinary panel in 2016 recommended in situ disease, preferably > 2 mm on resection margins, to achieve a reduced risk of local recurrence if accompanied with radiotherapy [45]. This issue represents a source of lively debate in several specific studies, without conclusive results about oncological outcomes in relation to margin width in DCIS [46,47]. 

To date, BCS is the standard treatment for DCIS lesions without evidence of the multicentricity or great extent of the disease [48]. As for invasive BC, the balance between oncological safety and cosmetic results is achieved by the amount of breast tissue removed depending on the extent of radiologically detected DCIS lesion, often associated with microcalcifications, in relation to breast size, individual clinical and pathological parameters, and patient preferences (see Figure 2). Positive margin and non-screening-detected DCIS were reported to be significantly associated with a higher risk of invasive recurrence in DCIS women in a meta-analysis studying the role of predictive LR factors [49]. A recent study analysing 2049 women treated by BCS, of which 1073 were with radiotherapy, after a median follow-up of 14 years, confirmed BCS + radiotherapy as a feasible option for women with large DCIS size (>40 mm), provided that negative margins can be achieved, and the addition of radiotherapy boost to further reduce the risk of recurrence [50]. The oncoplastic approach is essential in this setting as an oncologically safe tool for obtaining optimal aesthetic outcomes after BCS, performed in selected clinical conditions [51].

#### 3.1.4. Technical Features

The improvement of women’s physical and psychological well-being has been the primary purpose in the evolution of breast cancer surgery, favouring increasingly less disfiguring and radical approaches: adhering to the belief that “less is more, bigger is not better” [33], breast surgery has developed into an ever-increasing impulse towards greater respect for the quality of life of women, ensuring an excellent prognosis.

The value of the new definition of adequate margins, and the implication of molecular subtypes and of oncoplastic techniques, are extending the indications to BCS, such as in ipsilateral breast cancer recurrence or after neoadjuvant treatment [33]. Similarly, BCS for the treatment of DCIS is an approach considered oncologically safe, if free margins are guaranteed. An Italian Network of Senology Centers, Senonetwork Italia, recommends specific techniques and preoperative procedures to achieve adequate free margins, especially for nonpalpable and clinically occult lesions, such as DCIS [52]. Procedures such as charcoal, metal wire, and radio-guided occult lesion localisation (ROLL) can specifically support locating and removing the lesion. Routine skin removal during BCS for DCIS should be avoided, except in the presence of radiological signs of suspected large invasive components just beneath the skin or if required as planned by the oncoplastic team. An intraoperative specimen imaging evaluation was generally suggested in case of microcalcifications or the presence of clips left in the site of pre-operatory VABB, performing a 2D X-ray of the specimen to assist in obtaining macroscopically negative margins [52].

### 3.2. Mastectomy

#### 3.2.1. When to Perform Mastectomy in Ductal Carcinoma In Situ

Mastectomy is advised if DCIS is too extensive to allow breast conservation. Indeed, after a DCIS diagnosis, most women are eligible for BCS, while some may need mastectomy based on the extent of DCIS in the breast [40], also in relation to the breast size/DCIS extent ratio (see Figure 2). Furthermore, it represents a choice of treatment for women who choose to undergo this procedure over BCS; indeed, the choice of local treatment does not impact disease-related OS. Therefore, the individual’s preferences for risk reduction should be considered in the multimodal approach of DCIS management. The LR rate after mastectomy for DCIS has historically been demonstrated as low, with a meta-analysis studying 1574 patients demonstrating an LR of 1.4% [29]. Nonetheless, the closed clinical traits of this disease make intraoperative identification of the lesions difficult and stress the importance of performing a mastectomy that is technically as complete and radical as possible.

#### 3.2.2. Technical Features

In recent decades, the concept of mastectomy has turned towards an approach that increasingly respects body image and oncological safety. Skin-sparing mastectomy (SSM) and nipple-sparing mastectomy (NSM) are conservative mastectomies that satisfy this axiom and represent the standard in cases of radical non-tissue-sparing indications. In SSM, all the breast tissue is removed, preserving most of the skin flaps but sacrificing the nipple–areola complex (NAC) that must be removed. NSM represents a technical innovation in radical surgery with the preservation of the nipple–areola complex as well as the skin [53], ensuring better cosmetic outcomes and a higher satisfaction rate compared with SSM [54]. NSM is considered a safe oncological approach, as demonstrated by several studies [53,55], adhering to international recommendations that consider the absence of cancer in the NAC as mandatory [12]. 

Patients treated with mastectomy are appropriate candidates for breast reconstruction; when mastectomy is performed in DCIS, immediate breast reconstruction must always be proposed to the patient. Close collaboration between breast and plastic surgeons is of utmost importance [53]. As previously described, skin incision for NSM differs in relation to surgical personalisation and several clinical features [56]. It is important that the breast surgeon should altogether remove all the mammary gland, dissecting the breast tissue after identifying the avascular plane represented by Cooper’s ligaments and maintaining an adequate amount of subcutaneous fat to avoid flap necrosis. Respecting the anatomical breast limits ensures better reconstructive outcomes. Dissection of the retroareolar area must be radical, performing a retroareolar sample with an intraoperative frozen section [53]. Tissue expander, implant, or autologous graft are the possible choices for immediate reconstruction after breast gland removal.

### 3.3. Axillary Surgery

#### 3.3.1. When to Perform Axillary Surgery in Ductal Carcinoma In Situ

Before the sentinel lymph node biopsy (SLNB) era, an axillary dissection (ALND) was performed contextually for breast surgical treatment for DCIS patients [57]. Ductal carcinoma in situ is a neoplastic proliferation of epithelial cells confined within the basement membrane of the breast ductal–lobular system, therefore without a metastatic lymphatic spread. In such context, and with respect to the results of a large observational study [58], SNLB does not appear indicated. On the other hand, where ALND may nowadays appear to be an “obsolete” procedure in the surgical treatment of DCIS, SLNB remains a controversial choice. The current clinical practice recommendation is to perform upfront SLNB only in selected patients with DCIS with a significant risk of an upgrade of the lesion at final pathology [59]. The risk includes a mass highly suggestive of invasive disease on radiological findings and physical examination or extensive DCIS greater than 5 cm at imaging or when mastectomy is indicated, as confirmed by the 2014 ASCO update recommendations [60] (see Figure 2). The appropriateness of upfront SLNB in DCIS still lacks a clear consensus among surgical oncologists; however, the latest NCCN recommendations strongly endorsed it in DCIS patients treated with mastectomy [12].

#### 3.3.2. Ductal Carcinoma In Situ Upgrade Prediction

The preoperative prediction of upgraded diagnosis to invasive cancer on definitive pathology could avoid unnecessary axillary surgery, including SLNB. The 2019 St Gallen Consensus Conference confirmed the role of high-grade DCIS in upstaging the risk of invasive cancer at the definitive surgery [24]. A recent prediction model has been profiled to assess the most significant clinicopathological features potentially able to predict the upgrade of DCIS to invasive carcinoma on final pathologic diagnosis. In multivariable analysis, suspicious axillary lymph nodes on ultrasound (odds ratio [OR], 12.16; 95% confidence interval [CI], 4.94–29.95; *p* < 0.001) and high nuclear grade (OR, 1.90; 95% CI, 1.24–2.91; *p* = 0.003) were correlated with underestimation. Cases with biopsy performed using vacuum-assisted biopsy (VAB) (OR, 0.42; 95% CI, 0.27–0.65; *p* < 0.001) and lesion in size < 2 cm on mammography (OR, 0.45; 95% CI, 0.22–0.90; *p* = 0.021) and MRI (OR, 0.29; 95% CI, 0.09–0.94; *p* = 0.037) were reported as less likely to be upgraded [61]. In addition, a Chinese study suggested the non-minimal role of tumour-infiltrating lymphocytes (TILs) in the progression of DCIS to DCIS with microinvasion (DCIS-Mi), especially in HER2 + BC [62]. 

Extensive data analysis of 178,762 patients with DCIS extracted between 2012 and 2018 from the American National Cancer Data Base was recently studied at the Mayo Clinic [63]. Authors reported an overall axillary surgery overtreatment in 38% of the cohort, which was higher in patients undergoing mastectomy than patients undergoing BCS (88% vs. 19%, *p* < 0.001), describing an axillary surgery decrease in BCS patients (21% in 2012 to 17% in 2018, *p* < 0.001), and a slight increase in mastectomy patients (86% in 2012 to 90% in 2018, *p* < 0.001). Younger patient age, larger tumour size, higher grade, and ER-negative status were identified as significant factors correlated with axillary surgery in multivariate analysis [63]. Additionally, a recent systematic review and meta-analysis of data on 4388 patients diagnosed with ductal carcinoma in situ suggested that there is a limited justification for a priori axillary SLNB in the context of DCIS, reporting the absolute and relative risk of metastatic sentinel lymph node (SLN) for DCIS to be less than 5% and 1%, respectively. The study emphasised the role of specific aggressive clinicopathological parameters, e.g., clinically palpable lesion, presence of comedonecrosis or high-grade DCIS on diagnostic core biopsy in guiding the decision-making for SLNB in DCIS management [64]. 

To date, an ongoing randomised phase III trial is proposing a delay in performing SLNB in patients undergoing surgery for DCIS. Indeed, the SentiNot 2.0 trial (NCT04722692) is currently randomising patients to either radioisotope (control) or superparamagnetic iron oxide (SPIO) tracing of the axillary nodes at delayed SLNB in patients with a preoperative diagnosis of DCIS [65]. The trial aims to investigate the use of SPIO nanoparticles as a tracer for delayed SLNB in patients where upfront axillary surgery is oncologically deemed unnecessary and should be avoided [65].

## 4. Microinvasive Ductal Carcinoma In Situ

DCIS-Mi could be considered as the “interim” stage from ductal carcinoma in situ in progression to invasive BC [11,66]. DCIS-Mi is defined as invasive BC ≤ 1 mm in size (microinvasion) [67,68]. Microinvasion is usually observed in association with DCIS; the diagnosis of DCIS-Mi can be found in about 5–10% of DCIS [62]. The current debate focuses on microinvasion behaviour, if it should be managed similarly to DCIS or whether it represents a true, albeit small, invasive disease. Furthermore, there is no consensus on the role of routine axillary surgical staging when a diagnosis of microinvasion has been made. There are, however, no specific national or international guidelines on whether to perform axillary lymph node examination in patients with microinvasion, reflecting the paucity of data [69].

### DCIS-Mi Prognosis and Surgical Treatment Compared with Ductal Carcinoma In Situ

Recent data specified that DCIS-Mi presents a nearly two-fold increase in mortality rate compared with DCIS, which does not exhibit microinvasion, albeit with a similar risk of BC mortality compared to patients with small invasive cancer (0.2–1.0 cm in size) [70]. 

A retrospective clinical study based on the surveillance, epidemiology, and end results (SEER) database enrolled a total of 525,395 node-negative invasive BC patients treated between 1990 and 2013, categorised according to the size of the invasive component with 161,394 women with “pure” DCIS (defined without invasive component), 13,489 with microinvasive carcinoma, 153,856 with invasive cancer (invasive component size = 0.2 to 1.0 cm), and 196,656 with invasive cancer (invasive component size = 1.1 to 2.0 cm). After a median follow-up of 7.7 years, 15,613 women died of BC, including 1837 women with pure DCIS (1.1%), 323 women with DCIS-MI (2.4%), 3661 women with DCIS-IDC and an invasive component size of 0.2 to 1.0 cm (2.4%), and 9792 women with DCIS-IDC and an invasive component size of 1.1 to 2 cm (5.0%). At 20 years, the actuarial rate of BC mortality was 3.8% for patients with pure DCIS, 6.9% for patients with DCIS-MI, 6.8% for patients with DCIS-IDC and an invasive component size of 0.2 to 1.0 cm, and 12.1% for women with DCIS-IDC and an invasive component size of 1.1 to 2 cm [70]. A previous similar comprehensive study using the SEER database on a U.S. population with a median follow-up of 91 months reported that patients with DCIS-Mi had worse cancer-specific survival (CSS) (hazard ratio [HR], 2.475; *p* < 0.001) and OS (HR, 1.263; *p* < 0.001). In the multivariable analysis, microinvasion was described as an independent prognostic factor for worse CSS (HR, 1.919; *p* < 0.001) and OS (HR, 1.184; *p* < 0.001). Authors found that the 20-year cancer-specific mortality rate was 4.00% in ductal carcinoma in situ and 9.65% in DCIS-Mi (HR, 2.482; *p* < 0.001), highlighting that DCIS-Mi was associated with more aggressive biological parameters such as ER negative, PR negative, HER2 positive and lymph node metastasis (all *p* < 0.001) [71]. However, a meta-analysis of axillary staging in patients with ductal carcinoma in situ and microinvasion, which included 2959 patients from 23 studies, showed a survival rate of patients with microinvasion overall very similar to those with pure DCIS [72].

Recent findings suggested that the surgical approach of DCIS-Mi can be similar to pure DCIS, as mandatory axillary staging surgery. Such results also reported a group of patients with triple-negative DCIS or DCIS-Mi more associated with high rates of tumour recurrence, especially invasive recurrence [73].

Current guidelines from the National Comprehensive Cancer Network (NCCN) recommend treating patients with microinvasive carcinoma following surgery guidelines for ductal carcinoma in situ. Indeed, DCIS-Mi should refer to the DCIS margin definition when evaluating the adequate margin width (>2 mm), given that the majority of DCIS-Mi comprises DCIS, and systemic therapy application for this lesion more closely reflects the treatment pattern for DCIS than for invasive carcinoma [12]. The consequent overall breast surgery decision-making adheres to DCIS guidelines [73] (see Figure 2).

However, clinical outcomes of DCIS-Mi are not well characterised, and no consensus has been reached on what should be the most appropriate treatment, i.e., whether it should be treated as a stage 0 DCIS lesion or as a small invasive carcinoma [66,74]. In addition, the role of pathological and biological features on the prognosis and management of DCI-Mi is still unclear [75]. Findings from a study by Liu et al. suggest that DCIS-Mi represents a distinct entity, describing a significant association with a high nuclear grade, large tumour size, and comedonecrosis. DCIS-Mi was furthermore significantly more associated with high nuclear grade, large tumour size, comedonecrosis, absence of steroid receptors, HER2 overexpression, and high Ki67 index (*p* < 0.05) compared with DCIS [76]. These findings agree with a retrospective analysis, which pointed out that the distinct biological entity of DCIS-Mi, appears more similar to invasive disease [77]. 

A recent meta-analysis of 26 studies suggests that DCIS-Mi may show more aggressive biological and clinical behaviour than pure DCIS, reporting disease-free survival and loco-regional recurrence-free survival significantly shorter in patients with DCIS-Mi than in those with DCIS (hazard ratio, 1.52; 95% confidence interval, 1.11–2.08; *p* = 0.01, and hazard ratio, 2.53; 95% confidence interval, 1.45–4.41; *p* = 0.001, respectively) [78]. It underlined that a larger lesion size, axillary lymph node metastasis, comedonecrosis, poor grading, ER-negativity, PR-negativity, and HER2-positivity were significantly more frequently observed in association with DCIS-Mi than with pure DCIS. Thus, axillary surgery for DCIS-Mi still represents a conundrum; SLNB in DCIS-Mi is currently not well defined, while the rate of axillary metastases has been reported to be low (0–20%) [14,72,79]. 

The role of SLNB in clinically node-negative DCIS-Mi should be clearly defined; the possible future perspective could be to further de-escalate axillary surgical treatment in selected DCIS-Mi cases, to improve quality of life with no impact on women’s prognosis. Given the heterogeneous limitations of literature data, performing a risk assessment is advisable for guiding appropriate treatments, considering that for DCIS-Mi data are limited, and very often DCIS-Mi is the upstaged histological finding at definitive pathology after surgery for DCIS diagnosis.

The challenge in DCIS-Mi is to reach a balance between the risks of overtreatment versus undertreatment, and future research has the task of providing solid results that allow outlining rigorous recommendations in DCIS-Mi management, especially in axillary surgery strategy.

## 5. Conclusions and Future Directions

**Figure 3:**
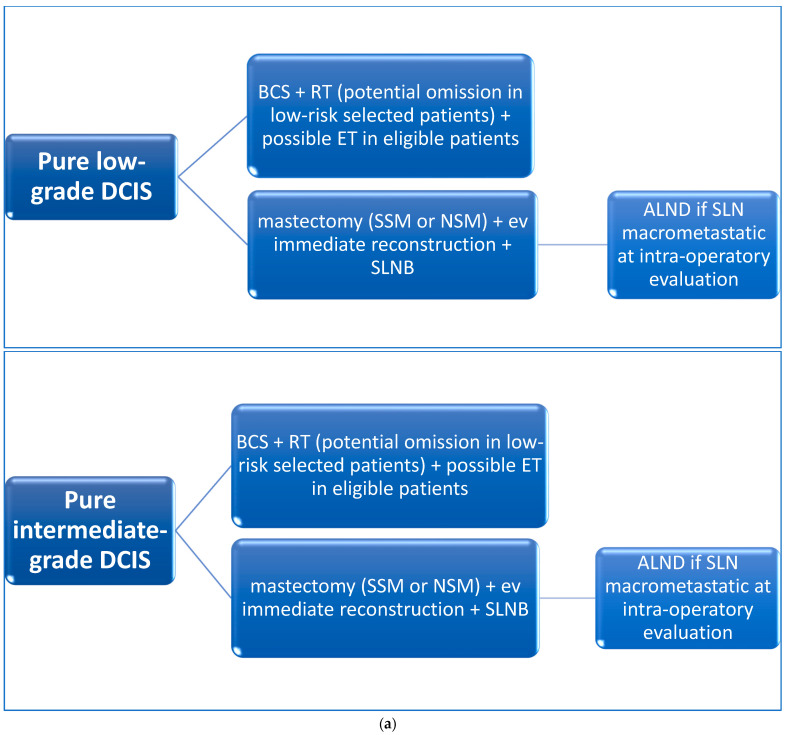

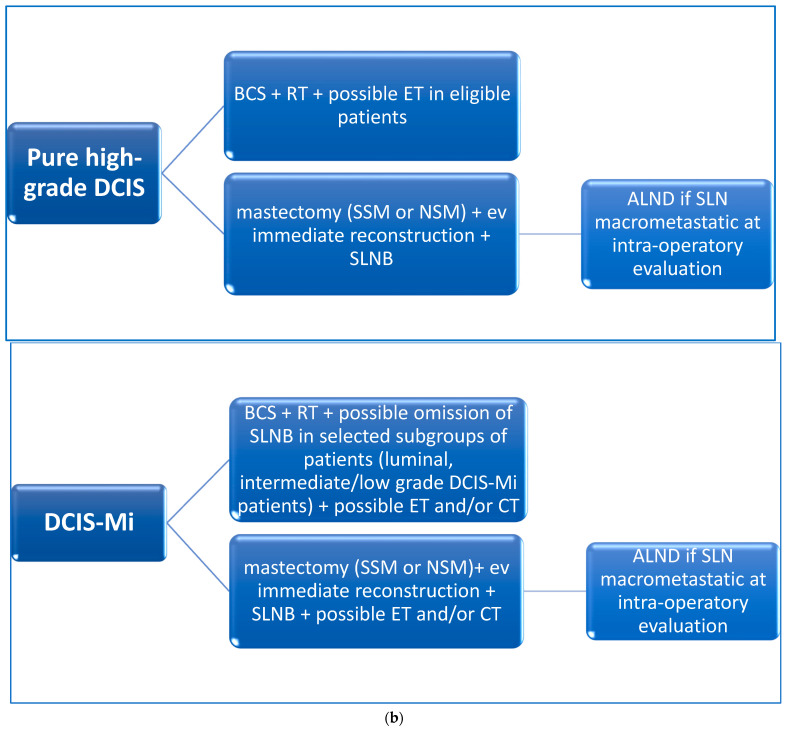
(**a**) DCIS and DCIS-Mi possible treatment options. DCIS—ductal carcinoma in situ; DCIS-Mi—microinvasive DCIS; BCS—breast-conserving surgery; ET—endocrine therapy; SLNB—sentinel lymph node biopsy; ALND—axillary lymph node dissection; SLN—sentinel lymph-node; RT—radiotherapy; CT—chemotherapy; SSM – skin sparing mastectomy; NSM – nipple sparing mastectomy. (**b**) DCIS and DCIS-Mi possible treatment options. DCIS—ductal carcinoma in situ; DCIS-Mi—microinvasive DCIS; BCS—breast-conserving surgery; ET—endocrine therapy; SLNB—sentinel lymph node biopsy; ALND—axillary lymph node dissection; SLN—sentinel lymph-node; RT—radiotherapy; CT—chemotherapy; SSM—skin sparing mastectomy; NSM—nipple sparing mastectomy.

The current DCIS and DCIS-Mi comprehensive treatment strategy scenario appears dynamic and heterogeneous (See Figure 3a,b). In the current era of precision medicine, considering the growing value of pathological, molecular, and genomic peculiarities in defining the prognosis of BC, including the setting of DCIS and DCIS-Mi, the research view should always focus on predicting the selected class of risk, for guiding breast surgeons in clinical practice, recognizing the future perspective of soft computing technologies and machine learning algorithms’ role in clinical decision-making [80]. 

The goal is to choose a better care strategy and minimise unnecessary overtreatments, preventing the related unfavourable effects and promoting an improvement in patients’ well-being and quality of life.

## Data Availability

Requests for research data may be addressed to Francesca Magnoni.
